# Alcohol Consumption among Freshman College Students in Spain: Individual and Pooled Analyses of Three Cross-Sectional Surveys (2005, 2012 and 2016)

**DOI:** 10.3390/ijerph18052548

**Published:** 2021-03-04

**Authors:** Alicia Busto Miramontes, Lucía Moure-Rodríguez, Narmeen Mallah, Ainara Díaz-Geada, Montserrat Corral, Fernando Cadaveira, Francisco Caamaño-Isorna

**Affiliations:** 1 Department of Public Health, University of Santiago de Compostela, 15782 Santiago de Compostela, Spain; alicia.busto@rai.usc.es (A.B.M.); narmeen.mallah@usc.es (N.M.); ainara.geada@usc.gal (A.D.-G.); francisco.caamano@usc.es (F.C.-I.); 2 Health Research Institute of Santiago de Compostela (IDIS), 15706 Santiago de Compostela, Spain; montse.corral@usc.es (M.C.); fernando.cadaveira@usc.es (F.C.); 3 Epidemiology and Public Health Networking Biomedical Research Centre (CIBERESP), 28029 Madrid, Spain; 4 Department of Clinical Psychology and Psychobiology, University of Santiago de Compostela, 15782 Santiago de Compostela, Spain

**Keywords:** heavy episodic drinking, risky alcohol consumption, freshmen

## Abstract

*Objective*: We aimed to evaluate changes in the prevalence of Heavy Episodic Drinking (HED) and Risky Consumption (RC) in freshman college students between 2005, 2012 and 2016; and to identify the explanatory variables of these patterns of consumption using individual and pooled analyses. *Methods*: A cross-sectional study involving 5260 students was carried out in Spain in 2005, 2012 and 2016. HED and RC were determined using the Alcohol Use Disorders Identification Test. Another questionnaire was used to measure parental education level and alcohol use, alcohol-related problems, age of onset of alcohol use and alcohol-related expectancies. Adjusted Odds Ratios (ORs) of RC and HED and their 95% Confidence Intervals were estimated using logistic regression. *Results*: An increase in the prevalence rates of HED and RC was observed among women during the three-study periods, nonetheless there was no statistically significant difference in the prevalence rates among men. High maternal educational level, living away from parental home, initiating drinking before the age of 15 and having positive expectancies about drinking are associated with higher prevalence of RC in both genders. High positive expectancies and early onset of alcohol use are associated with higher rates of HED among men and women. Students recruited in 2012 and 2016 are protected against RC in comparison to those recruited in 2005. *Conclusions*: The age of alcohol consumption onset is the most influencing factor on HED and RC for both genders in the three-study periods. Alcohol prevention campaigns targeting youth at early ages can reduce risky drinking behaviors.

## 1. Introduction

Alcohol consumption is a major public health problem that has reached epidemic levels and affects young people in particular [1,2]. The most common patterns of alcohol consumption among young people are: Heavy Episodic Drinking (HED) defined as a way of drinking that causes a rise in the Blood Alcohol Concentration (BAC) to at least 0.08% during a single drinking session (NIAAA, 2004) [3], and Risky Alcohol Consumption (RC) defined as drinking at levels that cause negative health consequences for the drinker or anyone else when this pattern of consumption persists over time [4].

Early adulthood represents a sensitive period for alcohol and drug use because important physical, intellectual, and social changes that shape the maturity and the development of an individual occur during this stage [5]. Alcohol misuse is associated with neurocognitive alterations in the developing brain, where changes in the brain continues to take place until late thirties [6,7]. Heavy alcohol consumption during youth is also associated with major social problems such as traffic accidents [8,9], unsafe sex [10], attempted suicides [11], poor academic achievement [12], and non-medical use of prescription drugs (NMUPD) [13].

Recent studies revealed that the age of entrance to the college is a determinant factor for alcohol-related problems because this stage is fundamental for the development of the individual and involves important social changes [14,15,16,17]. Furthermore, university students represent a highly vulnerable population due to academic stress, leaving the parental home, as well as the need for independence and social approval [14,15].

Early onset of alcohol consumption [18], prior drinking experiences [19], parental educational level [20], living away from parental home [21], positive alcohol expectancies [22], peer drinking [23] and pregaming [24] (i.e., drinking before going out) have been recognized as risk factors for the excessive use of alcohol and the misuse of other drugs among students [21,22,23,24]. Parental alcohol consumption and parental permissibility of alcohol use are also associated with student alcohol consumption [25,26,27]. These factors are very important because they do not only affect the current consumption behavior of the young freshman students but also influence their behavior in the future [21,28,29,30]. Moreover, the initial experiences of college drinking can predict future trajectories of alcohol consumption and affect students՚ success in college and subsequent adult roles and independence [21,22,23,24,25,26,27,28,29,30].

Gender differences in the frequency of drinking patterns and the explanatory role of other risk factors of drinking among females and males are still unclear. Social evolution like the increased prevalence of women studying at the university introduced changes in women’s role and consequently affected their pattern of alcohol consumption [31]. Moreover, the perception of the risk associated with drinking also varies by gender [32].

Several studies, mainly those carried out in USA, reported a reduction in the frequency of alcohol drinking patterns among the new generations of college students [33]. To the best of our knowledge, similar studies measuring changes in risky alcohol consumption between generations of college students in Spain are not available [34,35]. ESTUDES (Survey on drug use in Secondary Schools in Spain) [36], a study carried out in youths from 14 to 18, found a decrease in the prevalence of alcohol patterns from 43.4% to 36% in 2018, but this finding cannot be extrapolated to college students who are of a legal drinking age. Economic stability, alcohol regulations, law implementations, and university context differ considerably among countries [37]. In addition, college alcohol consumption and its associated factors can also vary between generations. Accordingly, there is a need for related studies in different sociocultural contexts.

Our research group has been monitoring alcohol consumption by university students in Galicia-Spain since 2005. We evaluated the patterns of consumption and the associated factors and determined the social consequences of drinking. So far, we have undertaken three cohort studies in freshman students in 2005, 2012 and 2016.

In the present study, we aimed: (1) to assess changes in the prevalence of HED and RC in college students between 2005, 2012 and 2016, and (2) to identify the explanatory variables of these patterns of consumption using individual and pooled analyses.

## 2. Materials and Methods

### 2.1. Study Design, and Population

A cross-sectional study was carried out in students of the University of Santiago de Compostela, Galicia, Spain. The database of the present study was constructed from the baseline data of three cohort studies that were undertaken in freshmen students of the same university in 2005, 2012, and 2016. The data was collected in the first semester of the corresponding academic year (between September and February). Participants were recruited using cluster sampling so that at least one of the first-year classes was randomly selected from each faculty/school of the university. In each faculty/school, the number of selected classes was proportional to that of the students. All freshman students who were present in the class on the day of the survey were invited to participate in the study (N_2005_ = 1382, N_2012_ = 1328 and N_2016_ = 2550). This study was approved by the Bioethics Committee of the University of Santiago de Compostela. The questionnaire was anonymous. Participants of the studies were informed both verbally and written, as part of the questionnaire, that the participation is voluntary, and that they can drop-out from the study at any time.

### 2.2. Data Collection

Each first-year classroom was visited by the researchers and all students attending the class were invited to participate in the study. In the three occasions, alcohol use was measured using the Galician validated version of the Alcohol Use Disorders Identification Test (AUDIT) [38,39].

In addition to the AUDIT, we used another questionnaire to determine factors potentially associated with alcohol use such as parental educational level and their alcohol use, alcohol-related problems, and age of onset of alcohol use.

This questionnaire also included an item that was specifically designed to measure alcohol-related expectancies. This question was generated using items from another questionnaire that was previously used in a population of young Spanish adults [40]. The freshman students of 2005 and 2012 were asked to rank 14 expectancies about the effects of alcohol. Subsequently, we computed the expectancies placed by the students in the top 7 positions by adding 1 point for each positive expectation and resting another point for each negative one as follows: it adds fun (+1), it helps me to socialize (+1), to feel more relaxed (+1), to forget about problems (+1), to endure problems (+1), it causes irritability (−1), anxiety (−1), depression (−1), confusion (−1), sleep-related problems (−1), nervousness (−1), aggression (−1), loss of control (−1), heaviness/drowsiness (−1). As for participants in the 2016 study, expectancies were measured using the Alcohol Expectancy Questionnaire—Adolescent, Brief (AEQ—AB) [41]. More details about data collection are available in reference number 21 [21].

### 2.3. Definition of Variables

#### 2.3.1. Independent Variables

Age of onset of alcohol consumption: participants were grouped into four categories according to the age of onset of alcohol use (after 16 years old, at 16, at 15, and before the age of 15).

Socio-demographic variables: gender, place of residence (in the parental home/away from the parental home), and maternal educational level (primary school/high school/university).

Score of alcohol expectancies: For the baseline data collected in 2005 and 2012, the number of positive and negative expectancies were used as described earlier to generate a score that ranged between −7 and 5, the maximum numbers of negative and positive expectancies, respectively. For the study of 2016, a score was generated from the Alcohol Expectancy Questionnaire—Adolescent, Brief (AEQ—AB) questionnaire [41]. For the analysis, all scores were divided into tertiles.

#### 2.3.2. Dependent Variable

Risky consumption: it was ascertained using a score generated from AUDIT [38,39]. According to the recommendations of the Galician validated version of the AUDIT [39], a gender-specific cut-off value was established. This variable was dichotomous and the adopted cut-off values for the score were: ≥ 5 for women and ≥ 6 for men.

Heavy drinking: It was determined using question no. 3 of the AUDIT: [38,39] “How often do you have six or more alcoholic drinks on a single occasion?” This question was answered by selecting one of the following options: Never, less than once a month, at least once a month, at least once a week, daily or almost daily. The answers “at least once a month, at least once a week, and daily or almost daily” were then grouped together to form “more frequently” category.

### 2.4. Statistical Analysis

The baseline data of the three previous cohort studies were pooled in the same database and analyzed using multilevel logistic regression models to obtain adjusted Odds Ratios (ORs) for risky consumption and heavy episodic drinking. Multilevel logistic regression models were chosen for this analysis because they are more flexible than traditional models and thus permits analyzing correlated data. The study-period was introduced into the model as a random variable.

Maximal models were generated at first by including all theoretical independent variables. The variables were excluded successively from the models if their elimination did not change the estimated OR by more than 10%. Data were analyzed using Generalized Linear Mixed Models in IBM SPSS Statistics for Windows, Version 20.0. Armonk, NY: IBM Corp.

## 3. Results

The response rate in the three studies reached 99.0% of students who were present in the class on the day of the survey. The samples collected in 2005, 2012 and 2016 are described in Table 1 and categorized by sex.

In relation to socio-demographic variables, the maternal educational level of male and female students had improved from 42.5% in 2005 to 52.7% in 2016. The distribution of students՚ residence (in or outside parental home) was not modified throughout the nine-years study period and nearly three quarters of students lived away from parents՚ home during their first year of university. The age of onset of alcohol consumption was delayed at least one year, for both women and men, from 15 to 16 years old.

Nearly 50% of students from both genders maintained a high-risk use of alcohol. Both patterns of consumption (HED and RC) remained stable in the three time periods for both sexes.

The relationship between the prevalence rates of RC and HED and the rest of socio-demographic variables are shown in Table 2. Having a mother with university education, living away from parents’ home, starting drinking before 15 and having positive alcohol expectancies were associated with higher prevalence rates of RC among males and females in the three studied study periods. However, only having high positive expectancies and starting alcohol consumption at early ages were related to higher rates of HED among men and women.

The association of socio-demographic variables and positive expectancies on RC and HED for the three study periods are shown in Table 3, Table 4 and Table 5.

### 3.1. Maternal Educational Level

The association of maternal educational level with patterns of alcohol consumption varied throughout the three-study periods. In 2005, maternal university education level was associated with higher risk of RC among male students [OR: 2.31 (95%CI: 1.14–4.75)] (Table 3). On the contrary, in 2012 and 2016, a lower maternal educational level (High school) was found to be associated with a two-fold increased risk of HED among men [OR: 1.99 (CI: 1.01–3.97)] (Table 4) and with higher odds of RC among women [OR:1.44 (CI: 1.05–2.00)] (Table 5).

### 3.2. Place of Residence

Living away from parents’ home during the first year of university is associated with increased risk of RC, though the association varied between males and females during the study periods. In 2005, female students who were residing away from parental home had double the risk of RC [OR: 2.34 (CI: 1.59–3.45)] and HED [OR:1.9 (CI: 1.2–3.2)], whereas in 2012, the place of residence was only associated with RC among males [OR: 2.6 (CI: 1.45–4.71)]. In 2016, both male and female students living outside parents’ home were at increased risk of RC [OR: 1.7 (CI: 1.1–2.5)] and [OR: 1.7 (CI: 1.2–2.3)], respectively.

### 3.3. Age of Onset of Alcohol Drinking Onset

The earlier the onset of alcohol consumption, (<15 years old), the higher the risk of RC and HED in both genders. This finding was observed in the three-study periods. The highest ORs were obtained for females recruited in 2016 who started drinking before the age of 15. These students were at more than 50 times higher risk of having risky consumption [OR: 52.03 (CI: 20.87–159.39)]. Male students who started drinking at 15 were also at 30 times higher risk of HED [OR: 30.46 (CI: 8.53–195.3)] in comparison with those who started drinking after the age of 16.

### 3.4. Alcohol Expectations

Students having positive alcohol drinking expectancies are at higher risk of RC and HED. Globally, the magnitude of the association had been decreasing from 2005 to 2016, where OR estimates of RC in women and men respectively declined from 6.14 and 5.97 in 2005, to 3.11 and 3.68 in 2012, and to 2.4 and 2.22 in 2016. OR of HED also dropped for women and men from 4.04 and 3.8 in 2012 to 1.57 and 2.27 in 2016, respectively.

### 3.5. Period-Based Analysis of the Pooled Data of 2005, 2012 and 2016

We examined the influence of study period (year) on both patterns of alcohol consumption (Table 6). Students belonging to studies carried out in 2012 and 2016 were at lower risk of RC and HED in comparison to students recruited in 2005. Early onset of alcohol consumption and positive expectancies of alcohol were confirmed as stable risk factors for both patterns of consumption in men and women. Having a mother with a secondary education level and living away from the parental home also remained as a risk factor for RC among males and females, although the place of residence did not have an influence on HED among males.

## 4. Discussion

The prevalence of HED and RC followed a stable trend in both genders. The effect of explanatory factors of alcohol consumption also presented some variations between 2005 and 2016. We found that early onset of alcohol consumption remained to strongly affect alcohol consumption patterns in 2016, while opposite findings were observed for having positive alcohol expectations. Maternal educational level and place of residence influenced several patterns of consumption among the three study periods. When participants of the three-time periods were analyzed together, we observed a reduced risk of RC for participants recruited in 2012 and 2016 in comparison with those of 2005. Living away from home, early alcohol consumption, having a mother with high school education level and positive alcohol expectations increased the risk of HED and RC in all male and female students of the three studies. The participation rate was around 98.0% in the three years, ensuring the representativeness of the studied population.

One of the most important differences between the three years is related to maternal educational level, that improved over time (42.5% in 2005 versus 52.7% in 2016 of mothers completing university education). This finding is in accordance with the increased ratio of women with college degrees in the general population [42]. The lower frequency of graduate women as compared to that of 2016, may be also related to the Spanish economic crisis in 2008 which made it difficult to youth people with low purchasing power to access the university. In previous studies, we considered this variable as a good indicator of economic status [34].

The age of onset of alcohol consumption delayed with years as reflected by the findings obtained from 2012 and 2016 studies (Table 1). This trend differs from the one reported by ESTUDES 2020 [36], in which the age of alcohol onset remained stable.

HED and RC reached high prevalence among freshman students. Nearly half of youths practiced RC with similar ratios between genders. Nonetheless, though HED prevalence was high in both genders, it was higher for male (one-third) than for female students (one-fifth). On the one hand, alcohol consumption in younger generations tends to be equal between genders, reflecting social changes that emerged in the last decade [31]. On the other hand, gender differences persist with respect to HED with higher prevalence among men than women. This finding could be explained by traditions and lower social permissibility to women to drink alcohol than men [43]. Another fact that could explain this gender difference, is that HED could be underestimated when measured using the third question of AUDIT test because that question does not allow differentiating between gender [38]. Our findings differ from that of ESTUDES 2020 which reported a decreasing trend of RC and HED patterns. These divergences can be explained by the specificity of our studied population (university students).

According to the literature, university students, who are of legal drinking age, are greater consumers of alcohol than their non-college peers [44]. Accordingly, it could be interesting to develop prevention campaigns for this subgroup of population.

Our results also do not coincide with that of studies from other countries which reported a decreasing trend of alcohol consumption patterns [33,36]. In our study, the prevalence rates of RC and HED remained stable over the years among males and females. These differences could be related to the duration of studies. In our study, we had a nine-year period (from 2005 to 2016), while in other studies the major changes in the trends of consumption were noticed in studies comparing data of 20-year intervals [33,45,46,47]. In this line, Wechsler et al., in a study that involved 119 colleges, found a negligible increase in the prevalence of heavy drinking pattern between 1993 (43.9%) and 2001 (44.4%) [45].

Concerning the place of residence, it seems that living away from parental home confers a higher risk of RC, but not of HED. Also, the influence of this variable varied according to the time-period where it affected only females in 2005, only males in 2012, and both genders in 2016. Living outside parents’ home, was related to RC in the United States. In specific, living in university campus increases the risk of alcohol consumption [47,48,49]. These behaviors could be partly explained by a less paternal supervision and a stronger peer influence among students who do not live in the family home [46,47,48]. Students living out of parental home might have a stable and probably daily alcohol consumption pattern compared to those living with their parents. A fact that could explain the lack of association between living outside parents’ home and HED is the cultural acceptance of alcohol consumption in Spain [50], even under greater paternal supervision, the effect of this variable is likely to be lower than that in other countries. The comparison between the three-time periods in our study showed that the place of residence influences both patterns of alcohol consumption in both genders, even though the effect is higher for RC.

It is noticeable the delay in the age of onset of alcohol use from the age of 15 to 16 in 2016. Considering that an earlier age of onset has been found as a risk factor for RC and HED patterns [51,52], this delay should protect students against risky drinking behaviors. The effect of the age of alcohol onset was high in the three study periods, but the greatest effect was observed in 2016. The fact that only 24.5% of women and 22.9% of men who participated in 2016 study had started drinking at the age of 15 or before (half the percentage found in 2005 and 2012), demonstrates that the age of onset depicts a subgroup of extreme personalities or behaviors.

For the three years, the age of onset was maintained as the most influencing variable on HED and RC among males and females. This finding reinforces that alcohol prevention campaigns at early ages can improve risky behaviors throughout youth. Nevertheless, our findings must be interpreted with caution because of the wide range of the corresponding confidence intervals.

In line with other studies and as expected, our findings reveal that positive alcohol expectancies represent a risk factor for alcohol consumption by males and females [22,34]. Nevertheless, the magnitude of the effect of having positive expectancies on RC and HED patterns of consumption is decreasing with time.

The lack of significant differences in the tendency of positive alcohol expectations between men and women reflects a reduction in gender inequality [31,53,54].

RC and HED trends remained stable over time. Although, when participants of the three-time periods were analyzed together and after adjusting for all confounding variables, RC seems to follow a downward trend in both genders as reflected by OR estimates (2005). This means that, for the same characteristics of subjects, the risk of practicing RC during the first year of university will decrease in the future. This tendency was not observed for HED.

Our study suffers from the following three limitations: (1) The data was collected by using self-administered questionnaires, which can lead to an under-or over-estimation of both independent and dependent variables [55]. Nonetheless, this is unlikely to occur as AUDIT is a validated test that was proved to produce reliable results when used by young adults and adolescents [56], and any misrepresentative data would probably affect descriptive rather than analytical findings [57]; (2) the most appropriate definition of HED in Spain implies differences between genders: more than five alcoholic drinks for women and more than six drinks for men, on a single occasion. The third question of the AUDIT therefore underestimates the prevalence of HED in women. However, this limitation will mainly affect descriptive and not analytical statistics. The use of a gender-specific instrument instead of AUDIT is recommended for future studies. (3) even though the dichotomization of the dependent variables facilitates the interpretation of the results, it can introduce noise to the analysis as not all items of the questionnaire will be able to effectively measure the target concept (i.e., alcohol consumption pattern).

Despite finding a high prevalence of alcohol consumption patterns among students belonging to 2005, 2012 and 2016, HED and RC remained stable over the study periods.

Important changes have been found in some explanatory factors of alcohol consumption such as delayed age of onset of alcohol use and decreased influence of positive alcohol expectancies. Further research is needed to assess this tendency during the next years as well as to adapt public policies to these changes.

## 5. Conclusions

HED and RC remained stable among males and females over the study periods. High positive expectancies and early onset of alcohol use are related to higher rates of HED and RC in both genders. The effect of early onset of alcohol use on consumption patterns is increasing with time, while the opposite is happening to the effect of positive expectations. Alcohol prevention campaigns at early ages can improve risky behaviors throughout youth.

## Figures and Tables

**Table 1:** Description of samples collected in 2005, 2012 and 2016.

	Percentage or Mean (95%CI)					
	Females			Males		
	2005 *n* = 992 (99.8%)	2012 *n* = 836 (97.0%)	2016 *n* = 1497 (90.1%)	2005 *n* = 371 (99.9%)	2012 *n* = 449 (95.1%)	2016 *n* = 864 (94.85%)
Maternal educational level						
Primary school	41.8 (38.4–45.3)	26.2 (22.5–30.1)	31 (28.2–33.9)	32 [ 26.5–37.8)	23.7 (18.8–29)	23.8 (20.4–27.5)
High school	33.6 (30.2–37.1)	34.9 (31.2–38.8)	26.4 (23.6–29.3)	27.6 (22.1–33.3)	30 (25.1–35.3)	23.5 (20–27.2)
University	24.6 (21.2–28.1)	38.9 (35.2–42.7)	42.5 (39.7–45.4)	40.3 (34.8–46.1)	46.3 (41.4–51.6)	52.7 (49.2–56.4)
Residence						
In parental home	24.7 (22.1–27.5)	20.4 (17.7–23.2)	20.1 (18.1–22.1)	29.7 (25.1–34.5)	24.5 (20.6–28.7)	23.9 (21.1 – 26.8)
away from parental home	75.3 (72.6 – 78)	79.6 (76.9–82.4)	79.9 (77.9–82)	70.3 (65.7–75.1)	75.5 (71.5–79.6)	76.1 (73.3–79)
Age of alcohol onset						
After 16 years old	16.5 (13–20.1)	12.8 (9.1–16.7)	14.1 (11.5–16.8)	23.4 (17.8–29.4)	15.2 (10.2–20.5)	15.2 (11.8–18.7)
At 16 years old	25.6 (22.2–29.3)	31.5 (27.8–35.4)	61.3 (58.7 –64)	21.6 (15.9–27.5)	26.6 (21.6 – 32)	61.9 (58.5–65.4)
At 15 years old	38.9 (35.4–42.5)	38.3 (34.6–42.2)	19.6 (17–22.3)	36.9 (31.2–42.8)	41.3 (36.3–46.6)	16.9 (13.5–20.4)
Before age of 15 years	19.0 (15.5–22.7)	17.4 (13.6–21.3)	4.9 (2.4–7.6)	18.1 (12.5–24.1)	16.9 (11.9–22.3)	6.0 (2.6–9.5)
AUDIT ^a^ score (mean)	5.42 (5.16–5.68)	5.19 (4.91–5.47)	5.78 (5.54–6.02)	7.65 (7.07–8.23)	6.78 (6.3–7.26)	6.75 (6.4–7.1)
Quest 3 AUDIT ^a,b^						
Never	61.2 (58.2–64.3)	55.5 (52.1–59.1)	54.4 (51.9–57.1)	39.1 (33.7–44.5)	38.2 (33.5–43.3)	38.1 (34.6–41.7)
Less than once a month	20.9 (17.8–23.9)	23.3 (19.9–26.8)	23.1 (20.5–25.7)	25.3 (19.9–30.7)	23.6 (18.9–28.7)	24.7 [(21.3–28.4)
Monthly	9.8 (6.8–12.9)	14.2 (10.8–17.7)	14.5 (12–17.2)	12.7 (7.3–18.1)	20.0 (15.3–25.1)	19.3 (15.8–22.9)
Weekly	8.1 (5–11.1)	6.8 (3.4–10.4)	7.9 (5.4–10.6)	22.9 (17.5–28.3)	18.0 (13.3–23.1)	17.8 (14.3–21.4)
More frequently	0.1 (0–3.2)	0.2 (0–3.8)	0.1 (0–2.7)	0.1 (0–2.7)	0.2 (0–5.3)	0.1 (0–3.8)
Risky Consumption	51.5 (48.3–54.8)	49.8 (46.3–53.4)	54.8 (52.2–57.5)	58.0 (52.8–63.2)	53.9 (49.2–58.8)	54.3 (50.9–57.8)
Heavy episodic drinking (%)	17.9 (15.6–20.3)	21.2 (18.5–24.1)	22.5 (20.4–24.7)	35.6 (30.7–40.7)	38.2 (33.7–43)	37.2 (33.9–40.6)

^a^ Alcohol Use Disorders Identification Test.; ^b^ How often do you have 6 or more alcoholic drinks on a single occasion?

**Table 2:** Prevalence rates of risky consumption (RC) and heavy episodic drinking (HED) by maternal educational level, residence, and age of onset of use of alcohol, and positive expectancies.

	Percentage of Subjects (%)											
	Risky Consumption						Heavy Episodic Drinking					
	Females			Males			Females			Males		
	2005	2012	2016	2005	2012	2016	2005	2012	2016	2005	2012	2016
Maternal educational level Primary school High school University	47.2 53.5 57.3 *	46.2 54.9 47.5	52.3 57.8 54.7^	47.4 60 65.8 *	50 53.5 56.3	52.3 57.1 53.4^	17.1 18.5 18.7	22.9 20.2 20.6	20.5 22.8 23.3	29.3 38 39	36.6 40.2 37.9	37.4 37.2 35.8
Residence In parental home Away from the parental home	42.2 54.9 *	47.6 50.5	47.8 56.3 *^	51.4 60.5	43.4 57.1 *	46.3 56.3 *	13.9 19.4 *	18.8 21.8	21.5 22.3	34.9 35.7	35.2 38.7	32 38
Age of onset of alcohol use After 16 years old At 16 At 15 Before the age of 15	30.5 57.3 69.2 78.9 *	26.2 47.2 71.2 78.1 *	10.4^ 39.8^ 65.6 80.6 *	32.8 60.2 73.9 89.3 *	36.8 53 76.6 75.4 *	15.2^ 36.4^ 66.5 79.3 *	7.9 13.4 28.5 38.7 *	7.8 15.1 32.8 47.9 *	6 10.2 26.9 39.8 *	17.2 31.4 46.4 66.7 *	26.5 32.3 57.9 62.7 *	4.3^ 19.4^ 43.9^ 68.1 *
Positive expectancies 1º tertile 2º tertile 3º tertile	27.5 55.7 70.8 *	26.9 53.5 63.6 *	42.9^ 55 68.1 *	35.4 64.5 76.2 *	31.7 60.4 66.4 *	44.8^ 52.3^ 68.4 *	6.9 18.9 24.3 *	8.7 17.1 36.7 *	16.7^ 22.8 28.3 *^	19.2 39.7 48.8 *	22.3 35.7 52.8 *	28.7 33.6 52.5 *
Total	51.5	49.8	54.8 ^	58	53.9	54.3	17.9	21.2	22.5 ^	35.6	38.2	37.2

* *p* < 0.05 among categories; ^ *p* < 0.05 among study periods.

**Table 3:** Influence of explanatory factors on Risky consumption and Heavy episodic drinking among male and female students in 2005. Results are presented as adjusted odds ratio (95%CI) ^a^.

	Females		Males	
	Risky Consumption	Heavy Episodic Drinking	Risky Consumption	Heavy Episodic Drinking
Maternal educational level				
Primary school	1(-)	1(-)	1(-)	1(-)
High school	1 (0.68–1.48)	0.95 (0.6–1.5)	1.60 (0.76–3.42)	1.09 (0.52–2.30)
University	1.38 (0.90–2.12)	1.01 (0.62–1.65)	2.31 (1.14–4.75)	1.64 (0.84–3.27)
Residence				
In parental home	1(–)	1(–)	1(–)	1(–)
Away from the parental home	2.34 (1.59–3.45)	1.93 (1.20–3.22)	1.5 (0.78–2.88)	0.72 (0.39–1.35)
Age of onset of use of alcohol				
After 16 years old	1(–)	1(–)	1(–)	1(–)
At 16	2.86 (1.8–4.59)	1.46 (0.73–3.13)	3.19 (1.45–7.21)	1.80 (0.75–4.7)
At 15	5.16 (3.09–8.75)	4.04 (2.06–8.57)	5.98 (2.42–15.62)	3.76 (1.47–10.45)
Before the age of 15	8.26 (4.6–15.21)	6.03 (2.99–13.07)	10.97 (4.11–32.18)	9.7 (3.75–27.53)
Positive expectancies				
1º tertile	1(–)	1(–)	1(–)	1(–)
2º tertile	3.31 (2.15–5.15)	2.97 (1.64–5.55)	2.11 (1.07–4.19)	2.06 (1.01–4.35)
3º tertile	6.14 (4.10–9.29)	3.47 (2.04–6.17)	5.97 (2.65–14.19)	4.01 (1.85–9.04)

^a^ Results of multivariate logistic regression adjusted for all variables included in the table.

**Table 4:** Influence of explanatory factors on Risky consumption and Heavy episodic drinking among male and female students in 2012. Results are presented as adjusted odds ratio (95%CI) ^a^.

	Females		Males	
	Risky Consumption	Heavy Episodic Drinking	Risky Consumption	Heavy Episodic Drinking
Maternal educational level				
Primary school	1(-)	1(-)	1(-)	1(-)
High school	1.32 (0.84–2.08)	0.66 (0.38–1.14)	1.73 (0.88–3.45)	1.99 (1.01–3.97)
University	0.95 (0.61–1.48)	0.79 (0.47–1.34)	1.52 (0.82–2.82)	1.11 (0.6–2.08)
Residence				
In parental home	1(-)	1(-)	1(-)	1(-)
Away from the parental home	1.23 (0.79–1.92)	0.91 (0.54–1.59)	2.6 (1.45–4.71)	1.41 (0.79–2.56)
Age of onset of use of alcohol				
After 16 years old	1(-)	1(-)	1(-)	1(-)
At 16	2.59 (1.54–4.47)	2.55 (1.1–7.01)	2.11 (1.06–4.32)	1.33 (0.63–2.91)
At 15	6.92 (3.98–12.36)	6.4 (2.81–17.32)	6.66 (2.99–15.54)	4.17 (1.89–9.65)
Before the age of 15	8.69 (4.25–18.61)	13.83 (5.55–39.97)	7.94 (3.08–22.14)	5.66 (2.27–14.92)
Positive expectancies				
1º tertile	1(-)	1(-)	1(-)	1(-)
2º tertile	2.57 (1.66–4.01)	1.72 (0.94–3.24)	2.83 (1.5–5.42)	1.5 (0.78–2.97)
3º tertile	3.11 (1.96–4.97)	4.04 (2.26–7.54)	3.68 (1.85–7.49)	3.8 (1.89–7.91)

^a^ Results of multivariate logistic regression adjusted for all variables included in the table.

**Table 5:** Influence of explanatory factors on Risky consumption and Heavy episodic drinking among male and female students in 2016. Results are presented as adjusted odds ratio (95%CI) ^a^.

	Females		Males	
	Risky Consumption	Heavy Episodic Drinking	Risky Consumption	Heavy Episodic Drinking
Maternal educational level				
Primary school	1(-)	1(-)	1(-)	1(-)
High school	1.44 (1.05–2.00)	1.23 (0.86–1.75)	1.3 (0.81–2.10)	1.03 (0.64–1.65)
University	1.23 (0.92–1.63)	1.27 (0.93–1.75)	1.14 (0.76–1.72)	1.02 (0.68–1.54)
Residence				
In parental home	1(-)	1(-)	1(-)	1(-)
Away from the parental home	1.67 (1.24–2.26)	1.16 (0.84–1.64)	1.68 (1.14–2.48)	1.36 (0.92–2.04)
Age of onset of use of alcohol				
After 16 years old	1(-)	1(-)	1(-)	1(-)
At 16	7.74 (3.26–22.89)	3.21 (0.92–20.34)	2.3 (0.96–6.15)	3.71 (1.01–24.02)
At 15	21.28 (9.21–61.87)	10.57 (3.26–64.86)	8.55 (3.86–21.72)	13.31 (3.97–82.82)
Before the age of 15	52.03 (20.87–159.39)	18.34 (5.47–114.2)	14.65 (5.92–40.75)	30.46 (8.53–195.3)
Positive expectancies				
1º tertile	1(-)	1(-)	1(-)	1(-)
2º tertile	1.51 (1.13–2.01)	1.36 (0.97–1.9)	1.01 (0.68–1.49)	0.98 (0.66–1.47)
3º tertile	2.40 (1.77–3.27)	1.57 (1.13–2.21)	2.22 (1.43–3.48)	2.27 (1.49–3.50)

^a^ Results of multivariate logistic regression adjusted for all variables included in the table.

**Table 6:** Influence of explanatory factors on Risky consumption and Heavy episodic drinking among all male and female students recruited in 2005, 2012 and 2016. Results are presented as adjusted odds ratio (95%CI) ^a^.

	Females		Males	
	Risky Consumption	Heavy Episodic Drinking	Risky Consumption	Heavy Episodic Drinking
Maternal educational level				
Primary school	1(-)	1(-)	1(-)	1(-)
High school	1.28 (1.03–1.58)	1.02 (0.79–1.3)	1.44 (1.02–2.02)	1.21 (0.87–1.70)
University	1.15 (0.93–1.41)	1.11 (0.88–1.4)	1.36 (1.01–1.84)	1.11 (0.82–1.50)
Residence				
In parental home	1(-)	1(-)	1(-)	1(-)
Away from the parental home	1.70 (1.38–2.08)	1.29 (1.01–1.65)	1.81 (1.36–2.41)	1.20 (0.90–1.60)
Age of onset of use of alcohol				
After 16 years old	1(-)	1(-)	1(-)	1(-)
At 16	3.00 (2.2–4.1)	1.97 (1.18–3.31)	2.43 (1.57–3.78)	1.7 (1.01–2.85)
At 15	7.12 (5.19–9.77)	5.4 (3.26–8.94)	7.05 (4.47–11.11)	4.67 (2.80–7.81)
Before the age of 15	12.58 (8.61–18.38)	9 (5.33–15.19)	11.2 (6.57–19.08)	9.89 (5.68–17.23)
Positive expectations				
1º tertile	1(-)	1(-)	1(-)	1(-)
2º tertile	2.05 (1.67–2.53)	1.65 (1.27–2.15)	1.47 (1.09–1.97)	1.22 (0.9–1.66)
3º tertile	3.28 (2.65–4.05)	2.28 (1.77–2.93)	2.9 (2.08–4.04)	2.78 (2.01–3.86)
Study periods				
2005	1(-)	1(-)	1(-)	1(-)
2012	0.71 (0.56–0.91)	1.25 (0.94–1.67)	0.71 (0.56–0.91)	1.25 (0.94–1.67)
2016	0.66 (0.53–0.83)	0.97 (0.76–1.25)	0.66 (0.53–0.83)	0.97 (0.76–1.25)

^a^ Adjusted by the all variables included in the column and random effect was considered among cohorts.

## Data Availability

All relevant data are included within the paper. Data contain potentially identifying information and sensitive participants information. For all these reasons, the authors must not upload the dataset to a stable, public repository. However, the authors agree to make freely available any materials and data described in the publication upon reasonable request to francisco.caamano@usc.es, principal investigator of this project and professor at University of Santiago de Compostela.
